# Association between viral hepatitis and increased risk of severe coronavirus disease 2019 (COVID-19) outcome: a systematic review and meta-analysis 

**Published:** 2022

**Authors:** Timotius Ivan Hariyanto, Claudia Jodhinata, Devina Adella Halim, Andree Kurniawan

**Affiliations:** 1 *Faculty of Medicine, Pelita Harapan University, Boulevard Jendral Sudirman Street, Karawaci, Tangerang, Indonesia *; 2 *Department of Internal Medicine, Faculty of Medicine, Pelita Harapan University, Boulevard Jendral Sudirman street, Karawaci, Tangerang, Indonesia*

**Keywords:** Hepatitis, Viral infection, Liver disease, Coronavirus, COVID-19

## Abstract

**Aim:**

The purpose of the current study is to analyze the potential association between viral hepatitis and the severity of COVID-19.

**Background:**

Coronavirus disease 2019 (COVID-19) is a worldwide concern that has created major issues with many aspects. It is important to identify the risk factors for severe outcomes of this disease. To date, no association between viral hepatitis and severe COVID-19 has not been established.

**Methods:**

Through November 5^th^, 2020, the databases of PubMed, Google Scholar, and medRxiv were systematically searched using specific keywords related to the focus of the study. All articles published on COVID-19 and viral hepatitis were retrieved. The Mantel-Haenszel formula with random-effects models was used to obtain the risk ratio (RR) along with its 95% confidence intervals (CIs) for dichotomous variables. The two-tailed *p*-value was set with a value ≤0.05 considered statistically significant. Restricted-maximum likelihood meta-regression was done for several variables, such as age, gender, hypertension, diabetes, and other liver disease.

**Results:**

Analysis results included a total of 16 studies with a total of 14,682 patients. Meta-analysis showed that viral hepatitis increases the risk of developing severe COVID-19 (RR 1.68 (95% CI 1.26 – 2.22), *p* = 0.0003, *I*^2 ^= 21%, random-effect modeling). According to the meta-regression analysis, the association between viral hepatitis and severe COVID-19 was not influenced by age (*p* = 0.067), diabetes (*p* = 0.057), or other liver disease (*p* = 0.646).

**Conclusion:**

An increase of severe COVID-19 risk is associated with viral hepatitis. To reduce the risk of COVID-19, patients with viral hepatitis should be monitored carefully.

## Introduction

 It has been more than one year since the world began suffering from coronavirus disease 2019 (COVID-19), but the number of positive cases and deaths continue to rise (1). This disease was declared a global pandemic and has not only affected health aspects, but also caused the world economy to plunge. Thus, it is important to carry out risk stratification, optimize the allocation of hospital resources, and direct public health recommendations and interventions (2, 3). Studies have identified hypertension, diabetes, dementia, anemia, cardiovascular disease, and thyroid disease as comorbidities associated with worse COVID-19 outcomes (4-12). Moreover, most chronic viral hepatitis infections, especially chronic hepatitis B and C infections, will develop into liver cirrhosis that can cause significant morbidity and mortality (13, 14). Previous studies have shown that liver cirrhosis is associated with undesirable prognoses in community and nosocomial-acquired pneumonia (15). Currently, there is no data regarding the association between viral hepatitis and COVID-19 outcomes. This article is aimed to provide evidence of the association between viral hepatitis and severe outcomes of COVID-19. 

## Methods

**Eligibility Criteria**

Several inclusion criteria were used in this review for assessing included studies: representation for clinical questions (P: positive/confirmed cases of COVID-19; I: a group of patients with viral hepatitis as their comorbidity; C: a group of patients without viral hepatitis; O: severe COVID-19), the study type of randomized control trial, cohort, clinical trial, case-cohort, and cross-over design, and availability of full-text article. Articles other than original research (e.g., review articles, letters, or commentaries), case reports, articles in languages other than English, articles on research in pediatric populations (17 years of age or younger), and articles on research in pregnant women were excluded.

**Search Strategy and Study Selection**

The literature search was performed on PubMed, Google Scholar, and medRxiv for articles published up to November 5th, 2020, using the keywords "viral hepatitis" or "clinical characteristics" or "risk factors" and "coronavirus disease 2019" or "COVID-19", and the search was limited to articles in English. The titles, abstracts, and full texts of all articles that met the search criteria were assessed, and those reporting the rate of fatty liver disease in COVID-19 patients with a clinically validated definition of "severe disease" were included in this meta-analysis. Investigation (forward and backward citation tracking) of the appropriate studies was also conducted to look for other potentially eligible articles. This study was conducted based on Preferred Reporting Items for Systematic Reviews and Meta-Analyses (PRISMA) guidelines (16).

**Data Extraction and Quality Assessment**

Two authors performed data extraction independently, and standardized forms that included author, year, study design, number of participants, age, gender, hypertension, diabetes, viral hepatitis, other liver disease (cirrhosis, liver failure, fatty liver disease), and severe COVID-19 were used. 

Severe COVID-19 was the outcome of interest. Severe COVID-19 was defined as patients who experienced (1) respiratory distress (≥30 breaths per min); (2) oxygen saturation at rest ≤93%; (3) ratio of the partial pressure of arterial oxygen (PaO2) to a fractional concentration of oxygen inspired air (FiO2) ≤300 mmHg; or (4) critical complication (respiratory failure, septic shock, and or multiple organ dysfunction/failure) or admission into ICU at the time of, or after, admission. 

The quality of the included cohort and case-control studies was evaluated by two investigators independently using the Newcastle-Ottawa Scale (NOS) (17). The selection, comparability, and exposure of each study were assessed, and studies were graded from zero to nine. Good quality studies were those that scored ≥7. 

**Statistical Analysis**

Review Manager 5.4 (Cochrane Collaboration) and Comprehensive Meta-Analysis version 3 software were used to perform the meta-analysis. Dichotomous variables were calculated using the Mantel-Haenszel formula with random-effects models. The I2 statistic was used to assess heterogeneity across studies, and values of < 25%, 26-50%, and > 50% were considered as low, moderate, and high degrees of heterogeneity, respectively. The effect estimate was reported as risk ratio (RR) along with its 95% confidence intervals (CIs) for dichotomous variables, respectively. The *p*-value was two-tailed, and statistical significance was set at ≤0.05. A restricted-maximum likelihood for pre-specified variables like age, gender, hypertension, diabetes, and other liver diseases (such as cirrhosis, liver failure, and fatty liver disease) was used to perform random effects meta-regression. To qualitatively assess the risk of publication bias, Begg’s funnel-plot analysis was performed. 

## Results

**Study Selection and Characteristics**

**Figure 1:**
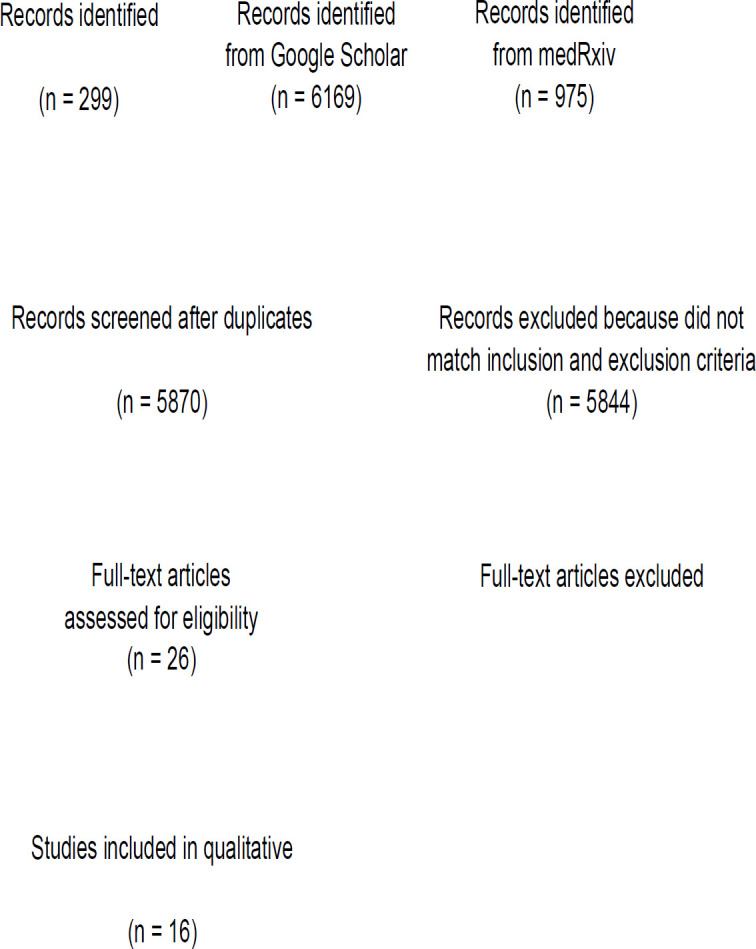
Preferred Reporting Items for Systematic Reviews and Meta-Analyses (PRISMA) flowchart

Through systematic electronic searches, a total of 6,468 records were obtained. A total of 5,870 records remained after the removal of duplicates. The titles/abstracts were screened, and a total of 5,844 records were excluded because they did not meet the inclusion criteria. The eligibility of 26 full texts was evaluated; 7 were excluded for not having a control/comparison group, and 3 were excluded because they did not mention the outcome of interest (severe COVID-19). Ultimately, a total of 16 studies were included in the meta-analysis (18-33) with a total of 14,682 COVID-19 patients (Figure 1). 

Of the 16 included studies, 15 were retrospective cohorts and one was a case-control study. Table 1 presents the summary of essential characteristics of the included studies. 

**Quality of Study Assessment**

Various study designs were included in this review, and appropriate scales or tools were used to assess them accordingly. The cohort and case-control studies were evaluated using the Newcastle Ottawa Scales (NOS) (Table 2). All of the studies included were rated as good.

**Viral Hepatitis and Outcome**

Pooled analysis showed a significant association of hepatitis with severe COVID-19, with low heterogeneity (RR 1.68 (95% CI 1.26 – 2.22), *p* = 0.0003, *I*2= 21%, random-effect modeling). 

**Figure 2:**
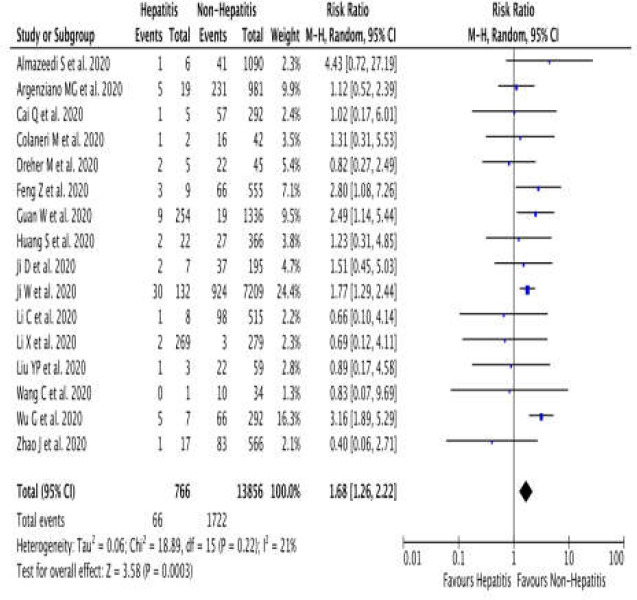
Forest plot that demonstrates the association of the viral hepatitis with severe coronavirus disease 2019 (COVID-19)

**Figure 3:**
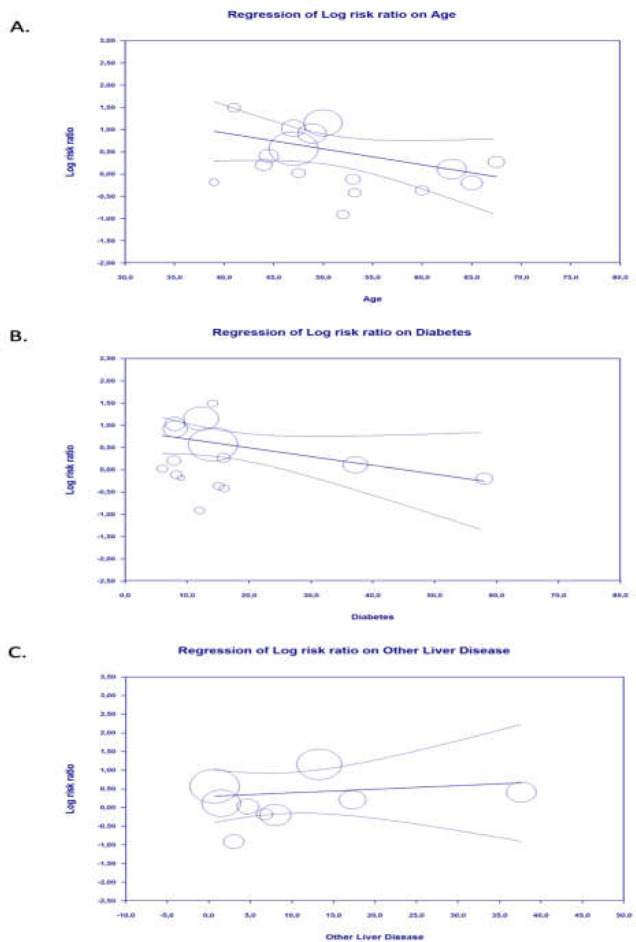
Bubble-plot for Meta-regression. Meta-regression analysis showed that the association between viral hepatitis and severe coronavirus disease 2019 (COVID-19) was not affected by age (A), diabetes (B), and other liver disease (C)

**Table 1:** Characteristics of included studies

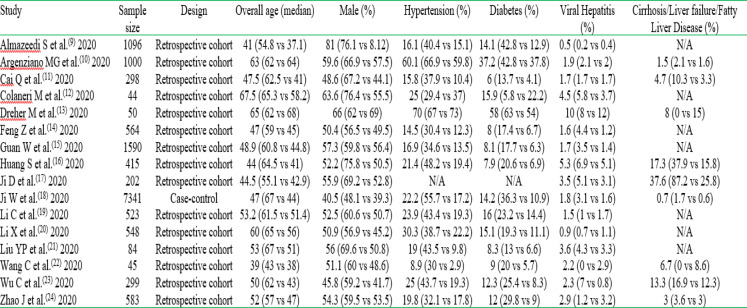

vs = Group A vs Group B

**Table 2:** Newcastle-Ottawa quality assessment of observational studies

First author, year	Study design	Selection	Comparability	Outcome	Total score	Result
Almazeedi S et al.^[9)^ 2020	Cohort	****	**	***	9	Good
Argenziano MG et al.^[10)^ 2020	Cohort	****	**	***	9	Good
Cai Q et al.^[11)^ 2020	Cohort	****	**	***	9	Good
Colaneri M et al.^[12)^ 2020	Cohort	**	**	***	7	Good
Dreher M et al.^[13)^ 2020	Cohort	***	**	***	7	Good
Feng Z et al.^[14)^ 2020	Cohort	***	**	***	8	Good
Guan W et al.^[15)^ 2020	Cohort	***	**	***	8	Good
Huang S et al.^[16)^ 2020	Cohort	**	**	***	7	Good
Ji D et al.^[17)^ 2020	Cohort	**	**	***	7	Good
Ji W et al.^[18)^ 2020	Case-control	***	**	***	8	Good
Li C et al.^[19)^ 2020	Cohort	***	**	***	8	Good
Li X et al.^[20)^ 2020	Cohort	***	**	***	8	Good
Liu YP et al.^[21)^ 2020	Cohort	***	**	***	8	Good
Wang C et al.^[22)^ 2020	Cohort	***	**	***	8	Good
Wu C et al.^[23)^ 2020	Cohort	****	**	***	9	Good
Zhao J et al.^[24)^ 2020	Cohort	***	**	***	8	Good

**Meta-Regression**

Based on meta-regression analysis, age (*p* = 0.067) (Figure 3A), gender (*p* = 0.449), hypertension (*p* = 0.089), diabetes mellitus (*p* = 0.057) (Figure 3B), and other liver disease (such as cirrhosis, liver failure, or fatty liver disease) (*p* = 0.646) (Figure 3C) did not affect the association between viral hepatitis and severe COVID-19. 

**Subgroup Analysis**

Subgroup analysis revealed a higher and statistically significant risk ratio (RR) for the association between viral hepatitis and severe COVID-19 in fully-published studies (RR 1.70 (95% CI 1.27 – 2.28), *p* = 0.0003, I2 = 18%, random-effect modeling) compared with pre-printed studies which showed non-statistically significant results (RR 1.35 (95% CI 0.42 – 4.40), *p* = 0.61, I2 = 55%, random-effect modeling).

**Publication Bias**

No publication bias was detected by funnel-plot analysis (Figure 4) regarding the association between viral hepatitis and severe COVID-19; analysis displayed a qualitatively symmetrical inverted funnel-plot.

## Discussion

The results of this comprehensive meta-analysis of 16 studies indicate that viral hepatitis is associated with severe COVID-19. Age, gender, hypertension, diabetes, and other liver diseases (such as cirrhosis, liver failure, and fatty liver disease) did not influence the association. Subgroup analysis revealed that the association between viral hepatitis and severe COVID-19 was statistically significant in fully-published studies but not in pre-printed studies. 

Several reasons can be proposed to explain the relationship between viral hepatitis and severe COVID-19. First, most chronic viral hepatitis infections will progress to the development of liver cirrhosis. In a study by Iavarone et al., liver cirrhosis itself was associated with high rates of 30-day mortality in COVID-19 patients, with 30-day mortality reaching 34% (95% CI 23 – 49), higher than in COVID-19 patients without cirrhosis (34). Moreover, studies showed that hepatitis B and hepatitis C infection could hamper the development of gut microbiota community, significantly decrease gut microbiota diversity, and therefore lead to dysbiosis (35, 36). A great proportion of COVID-19 patients that fall into the category of “severe disease” is related to the development of acute respiratory distress syndrome (ARDS), one of the most common complications in COVID-19 patients. Several experimental and clinical studies have suggested that gut microbiota play a key role in the pathogenesis of sepsis and ARDS through the gut-lung axis. Gut microbiota also play a part in the regulation of immune response, generally through T-regulatory cells (37, 38). Therefore, dysbiosis that happens in viral hepatitis will lead to the development of severe outcomes in many diseases, such as ARDS. Patients with viral hepatitis should be advised to take extra precautions to minimize the risk of exposure to the COVID-19 virus. Physicians should also engage in close monitoring of viral hepatitis patients with suspected COVID-19 for the timely detection of signs of disease progression. Finally, the presence of viral hepatitis should be regarded as an important factor in future risk stratification models for COVID-19.

The current study had several shortcomings. Foremost, the viral hepatitis medications and disease duration could not be analyzed because of the lack of data available in the included studies. Second, some pre-print studies were included as an effort to minimize the risk of publication bias; however, extra efforts were made by the authors to ensure that only sound studies were included. The authors anticipate that most of the studies currently available in pre-print form will eventually be published and identified through ongoing electronic literature surveillance. The authors hope that early insight into further risk stratification for COVID-19 can be provided by this research.

## Conflict of interests

The authors declare that they have no conflict of interest.
